# Complete Genome Sequence of Vibrio natriegens Strain PWH3a

**DOI:** 10.1128/mra.01108-22

**Published:** 2023-01-04

**Authors:** Kevin Xu Zhong, Amy M. Chan, Anwar Al-Qattan, Yueang Li, Curtis A. Suttle

**Affiliations:** a Department of Earth, Ocean and Atmospheric Sciences, The University of British Columbia, Vancouver, BC, Canada; b Department of Microbiology and Immunology, The University of British Columbia, Vancouver, BC, Canada; c Department of Botany, The University of British Columbia, Vancouver, BC, Canada; d Institute for the Oceans and Fisheries, The University of British Columbia, Vancouver, BC, Canada; University of Southern California

## Abstract

Vibrio natriegens strain PWH3a, isolated from the Texas Gulf Coast, is used as a model organism in marine microbiology. Here, we report the complete genome sequence of strain PWH3a, which has two circular chromosomes, 4,650 coding sequences, 34 rRNA, 4 noncoding RNA (ncRNA), 131 tRNA genes, and one Mu-like prophage sequence.

## ANNOUNCEMENT

Vibrio natriegens strain PWH3a was isolated in 1989 by swabbing seawater collected from the research pier in Aransas Pass at The University of Texas at Austin, Marine Science Institute (27.8380 N, 97.0504 W) onto CPM seawater plates (0.05% Casamino Acids, 0.05% Peptone and 1% agar in filtered seawater) (1, 2). Used as a model organism to study viruses in marine systems, mechanisms of viral decay, and iron sequestration (1–15), isolate PWH3a was initially described as *V. natriegens* strain PWH3a (3) and later as Vibrio alginolyticus strain PWH3a (13, 14), based on partial 16S rRNA gene sequences. Here, we report that analysis of the complete genome firmly places strain PWH3a as an isolate of Vibrio natriegens.

**FIG 1:**
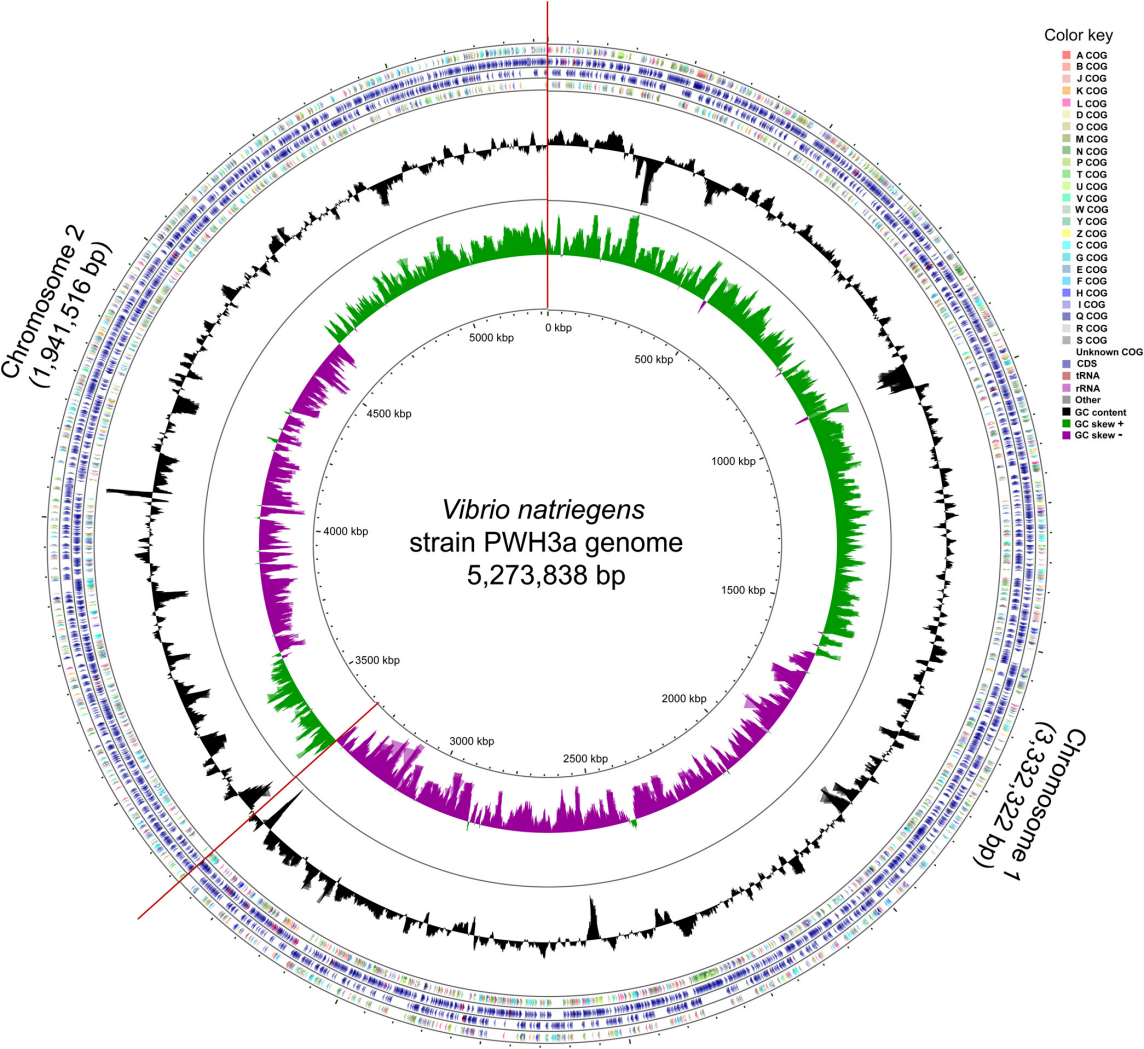
Circle map of the Vibrio natriegens strain PWH3a genome. The outermost to innermost rings of the map represent the following: (i) Cluster of Orthologous Gene (COG) functional categories for coding sequences of the forward strand; (ii) forward-strand sequence features; (iii) reverse-strand sequence features; (iv) COG functional categories for the reverse-strand coding sequences; (v) GC content, shown by the black ring; and (vi) GC skew, with the green and purple bands representing positive and negative values, respectively.

Single colonies streaked directly from a glycerol stock prepared in 1991 were used to start an overnight culture (CPM broth, 25 practical salinity units [PSU] seawater, 27°C, shaker incubator at 150 rpm). These cells were sent to MiGS (Pittsburgh, PA) for genomic DNA extraction (Zymo fungal/bacterial DNA miniprep kit; Zymo Research, Irvine, CA) and hybrid assembly sequencing (Small Nanopore Combo). The Illumina sample was prepared using the Illumina DNA library prep kit (Illumina, Inc., San Diego, CA) and sequenced on an Illumina NextSeq2000 instrument with 151-bp paired-end chemistry, generating 3,253,684 short reads. The Nanopore sample was prepared using the Oxford Nanopore Technologies (ONT, UK) ligation sequencing kit and sequenced on a MinION instrument using an R9 flow cell (R9.4.1), with base calling performed using ONT Guppy v.4.2.2, yielding 85,721 long reads. Adapters and low-quality reads were trimmed using bcl2fastq v2.19.0 (16) and Porechop v.0.2.4 (17) for Illumina and ONT sequences, respectively. Unicycler v.0.5.0 (18) was used to perform hybrid assembly of both read types. The complete bacterial genome consists of two circular chromosomes (3,332,322 bp and 1,941,516 bp) with an average GC content of 44.8% (Fig. 1). Analysis using CheckM v.1.0.18 (19) revealed that PWH3a genome was 99.74% complete and had 2.38% contamination compared to other *Vibrio* species reference genomes in the database.

The genome was annotated using NCBI Prokaryotic Genome Annotation Pipeline v.6.0 (PGAP) (20), which predicted 4,650 coding sequences and 34 rRNA, 4 noncoding RNA (ncRNA), and 131 tRNA genes. The isolate PWH3a was identified as *V. natriegens* based on taxonomic assignment of the overall genomic features using Kaiju v.1.8.2 (21), Kraken2 v.2.1.2 (22), CAT v.4.6 (23), PGAP v.6.0 (20), GTDB-Tk v.2.1.0 (24), and MetaErg v.1.2.3 (25).

Prophage sequences were identified using PHASTER (26) and VIBRANT v.1.2.1 (27), resolving one 26,631-bp putative prophage in chromosome 1 at the region from bp 382291 to 408922. This prophage, which appears to be a Mu-like phage (*Myoviridae*), contains phage integration sites, *attR* and *attL*, as well as genes encoding integrase, hydrolase, baseplate hub protein, transposase, and methyltransferase (Table 1).

**TABLE 1:** Annotation details of the 26,631-bp putative prophage sequences identified in chromosome 1 of Vibrio natriegens strain PWH3a using PHASTER

CDS^*a*^ position in chromosome	BLAST hit	*E* value
382291–382308	*attL*	0
393703–394890	PHAGE_Pseudo_PMGL (NC_076765): integrase; PP_00400; phage (gi374531680)	1.55E−23
395739–396281	PHAGE_Klebsi_ST76_0XA48phi5.4 (NC_049450): methyltransferase type11; PP_00401; phage (gi100001)	2.34E−15
Complement (396555–396863)	Hypothetical; PP_00402	0
Complement (397217–397292)	tRNA	0
397244–397261	*attR*	0
Complement (397445–399316)	PHAGE_Klebsi_ST13_OXA48phi12.1 (NC_049453): hypothetical protein; PP_00403; phage (gi100004)	0
Complement (399416–401173)	PHAGE_Klebsi_ST13_OXA48phi12.1 (NC_049453): hypothetical protein; PP_00404; phage (gi100003)	0
Complement (401317–401760)	PHAGE_Bacill_G (NC_023719): gp509; PP_00405; phage (gi593777964)	9.51E−14
Complement (401788–402003)	PHAGE_Klebsi_ST147_VIM1phi7.1 (NC_049451): hypothetical protein; PP_00407; phage (gi100043)	9.3E−37
402788–403249	PHAGE_Klebsi_ST13_0XA48phi12.1 (NC_049453): hypothetical protein; PP_00406; phage (gi100002)	0
403456–404205	PROPHAGE_Shewan_MR-1: ISSod6, transposase; PP_00408; phage (gi24374783)	1.51E−139
404273–405700	PHAGE_Mycoba_Milly (NC_026598): hydrolase; PP_00409; phage (gi764160985)	5.51E−09
Complement (405726–405737)	Hypothetical; PP_00410	0
405989–406348	Hypothetical; PP_00411	0
406525–406827	Hypothetical; PP_00412	0
406843–407646	Hypothetical; PP_00413	0
Complement (407702–408922)	PHAGE_Sinorh_phiM7 (NC_041929): baseplate hub protein; PP_00414; phage (gi100277)	9.04E−107

a CDS, coding DNA sequence.

Default parameters were used for all software. Genomic DNA was not size selected prior to library preparation for ONT sequencing.

### Data availability.

The genome sequence of *V. natriegens* strain PWH3a has been deposited in GenBank under accession no. CP107282 to CP107283. Raw sequencing reads were deposited under NCBI SRA accession no. SRR22423454 to SRR22423455. Strain PWH3a is available upon request from the corresponding authors.
